# Race and ethnicity and self-reported racial/ethnic discrimination in breast cancer patient interactions with providers in the Pathways Study

**DOI:** 10.1007/s10549-024-07499-0

**Published:** 2024-10-05

**Authors:** Kevin R. Bitsie, Thomas A. Pearson, Marilyn L. Kwan, Lusine Yaghjyan, Lisa Scarton, Salma Shariff-Marco, Lawrence H. Kushi, Ting-Yuan David Cheng

**Affiliations:** 1 Department of Epidemiology, University of Florida, Gainesville, FL 32610 USA; 2 Division of Research, Kaiser Permanente Northern California, Pleasanton, CA USA; 3 College of Nursing, University of Florida, Gainesville, FL USA; 4 Department of Epidemiology and Biostatistics, School of Medicine, University of California, San Francisco, San Francisco, CA USA; 5 Division of Cancer Prevention and Control, Department of Internal Medicine, The Ohio State University, Columbus, OH 43201 USA

**Keywords:** Breast cancer, Discrimination, Race/ethnicity, Patient-provider, Institutional racism, Breast cancer disparities

## Abstract

**Purpose:**

To examine the association of race and ethnicity groups with self-reported racial/ethnic discrimination in patient-provider interactions during the diagnosis and treatment for breast cancer.

**Methods:**

We analyzed data from the Pathways Study, a prospective cohort of women diagnosed with breast cancer from 2006–2013 in the Kaiser Permanente Northern California Health Care System. Racial/ethnic discrimination in patient-provider interactions was assessed with two questions from the Interpersonal Processes of Care survey at baseline and 6-months and 24-months post-diagnosis. Logistic regression was performed to compare women who self-identified as racial or ethnic minorities with Non-Hispanic White (NHW) women. Covariates included age at diagnosis, country of origin, education level, income, marital status, and medical provider’s race/ethnicity.

**Results:**

Our sample included 1836 participants: 1350 NHW women and 486 women (87 Black, 208 Asian American, 153 Hispanic, 38 American Indian/Alaskan Native/Pacific Islander [AIANPI]) from racial or ethnic minority groups. In multivariate analysis, minority women were more likely to report racial/ethnic discrimination in patient-provider interactions than NHW women (adjusted odds ratio [aOR]: 4.73; 95% confidence interval [CI] 3.45–6.50). Specifically, Black women were most likely to self-report racial/ethnic discrimination in patient-provider interactions (aOR: 9.65; 95% CI 5.92–15.70), followed by Asian (aOR: 5.39; 95% CI 3.46–8.40), Hispanic (aOR: 2.55; 95% CI 1.54–4.14), and AIANPI (aOR: 1.74; 95% CI 0.58–4.25) women, compared with NHW women.

**Conclusion:**

Racial/ethnic discrimination was more likely self-reported from minority women diagnosed with breast cancer. Additional studies are needed to understand the mechanisms and impact of racial/ethnic discrimination in patient-provider interactions on disparities.

**Supplementary Information:**

The online version contains supplementary material available at 10.1007/s10549-024-07499-0.

## Introduction

Non-Hispanic White (NHW) women are more likely to be diagnosed with breast cancer than Black women; however, age-adjusted breast cancer mortality rates are highest among Black women than among all other racial/ethnic groups, with mortality rates exceeding NHW women by 40% [1–3]. Differences in reported prognostic factors frequently associated with breast cancer mortality, such as late-stage diagnosis and diagnosis with triple-negative breast cancer, do not fully explain this long-standing disparity [4, 5]. Thus, other factors beyond screening, clinical characteristics, and tumor biology could contribute to these differences.

Interpersonal aspects in patient-provider relationships have important influences on treatment decisions related to the timely receipt of evidence-based cancer care options and improved quality of life outcomes [6–8]. The importance of trust in providers and the healthcare system has significant implications for patient satisfaction, especially among Black patients who report experiencing healthcare discrimination in medical settings [9, 10]. Another study found that Black patients had the highest reporting of discrimination in medical settings irrespective of attribution [11]. Additionally, discrimination in medical settings was significantly associated with healthcare quality outcomes (i.e., fair/poor quality of care, not involved in decisions, and little time with doctor) [11].

However, previous research examining self-reported racial/ethnic discrimination in patient-provider interactions and outcomes has been limited by sample size [11, 12], unknown provider race or ethnicity [12], and cross-sectional study design [9, 11–13]. Lis et al., reported interpersonal factors and patient-provider relationships as key predictors of patient satisfaction in oncology patients [14]. Implicit racial bias on the part of providers can affect patient-provider communication and patient-treatment decisions [15]. As the patient-provider relationship is essential to facilitate optimal care management and treatment plans, racial or ethnic discrimination could affect the initiation and adherence to treatment plans and subsequent morbidity and mortality.

In this study, we analyzed data from the Pathways Study, a large prospective cohort study of breast cancer survivors with detailed baseline and follow-up information on clinical and social measures, to identify self-reported racial/ethnic discrimination in patient-provider interactions. We hypothesized that women who self-identified as racial or ethnic minorities were more likely to report racial or ethnic discrimination in patient-provider interactions than their NHW counterparts.

## Methods

### Study population

The Pathways Study is a prospective cohort study of 4504 women diagnosed with and treated for invasive breast cancer from 2006 to 2013 in a large, integrated health care system, Kaiser Permanente Northern California (KPNC). Eligibility included being at least 21 years old at the time of their breast cancer diagnoses, having no prior history of cancer besides non-melanoma skin cancer, and being members of the KPNC integrated health care delivery system. Cancer diagnoses were ascertained through pathology reports and later confirmed by medical record review (Supplemental Fig. 1) [16]. The Institutional Review Board (IRB) at KPNC and University of Florida approved these secondary analyses.

**Fig. 1:**
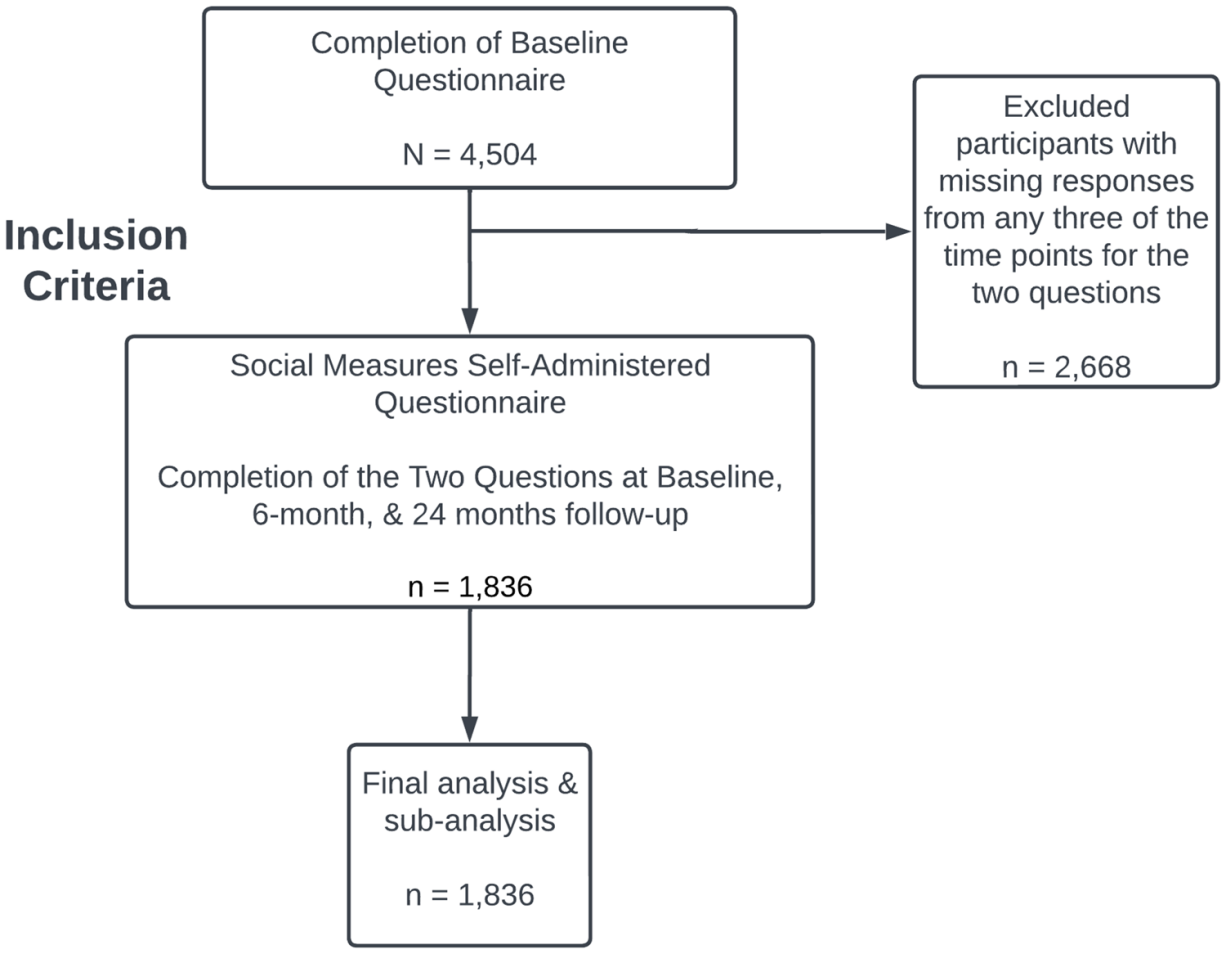
Inclusion criteria for analysis of self-reported racial/ethnic discrimination in patient-provider interactions in the Pathways Study, for participants with complete data at baseline, and 6-months, and 24-month follow-ups (*n* = 1836)

Data were collected from participants during a baseline interview (on average about two months post-diagnosis); mailed follow-up questionnaires at 6 and 24 months post-diagnosis; and ongoing bi-annual telephone interviews. Among the various items ascertained, baseline surveys included information on sociodemographic variables, health history, diet, physical activity, quality of life, and psychosocial measures. Sociodemographic and health history data included self-identified race and ethnicity, age, education, income, and marital status [16].

### Discrimination assessment

Among the 4504 enrolled participants in the Pathways Study, participants with data related to discrimination in patient-provider relationships assessed at baseline, and at 6-months and 24-months follow-up were included. These two questions were: “How often did doctors pay less attention to you because of your race or ethnicity?” (*n* = 1838), and “How often did you feel discriminated against by the doctors because of your race or ethnicity?” (*n* = 1854). These questions are components of the Interpersonal Processes of Care (IPC) survey, an 18-item questionnaire assessing patient-provider interactions in various domains related to the quality of care within the past 12 months. Domains within the IPC survey include: lack of clarity, elicited concerns, explained results, compassion, patient-centered decision-making, discrimination due to race/ethnicity, and disrespectful office staff [16, 17]. The IPC survey has been validated in diverse racial and ethnic backgrounds and the discrimination due to race/ethnicity domain had an internal-consistency reliability of 0.77 [13]. Two prior Pathways studies analyzed a different definition of self-reported racial or ethnic discrimination in patient-provider interactions, which were based solely on the baseline questionnaire [13, 18]. We further extended these analyses to examine patient-provider interactions beyond the time of breast cancer diagnosis by using data from the IPC survey at baseline as well as 6-month and 24-month follow-up. Our goal was to capture more information during breast cancer treatment and to further explore interactions between patients and providers.

The responses for these questions related to discrimination due to race/ethnicity domain were recorded on a Likert scale ranging from 1 = ‘Never’ to 5 = ‘Always.’ For each question, we combined all responses from the baseline and two follow-up surveys into two options–Never or Ever. “Ever” self-reported racial/ethnic discrimination in patient-provider interactions was defined as a response of “Rarely,” “Sometimes,” “Usually,” and “Always” at any time point, while “Never” was defined as a response of “Never” at all three time points of the IPC survey. Finally, we combined these two questions to expand self-report of racial/ethnic discrimination in patient-provider interactions. Participants missing responses from any three of the time points for the two questions were excluded (*n* = 2668) (Supplement Table 1). As a result, 1836 were included in the final analysis. Additional sub-analysis stratified by five race and ethnicity groups: Hispanic, non-Hispanic Black, non-Hispanic Asian, American Indian/Alaska Native/Pacific Islander and NHW women as the reference group. Participants who self-reported their race as American Indian, Alaska Native, or Pacific Islander (AIANPI) were combined due to small numbers (*N* = 38), providing a total of 1836 participants (Fig. 1). In addition, we categorized covariates including country of origin (US born and foreign-born), highest education level completed (high school or less, some college, college graduate, post-graduate, and Unknown), household income level (< $25 K, $25 K–$49 K, $50 K–$89 K,  ≥ $90 K, and Unknown), marital status (Married/Lived as Married, Single/Separated/Divorced/Widowed, and Unknown), and reported provider race or ethnicity (Asian, Black, Hispanic, White, and Unknown) of their principal provider of oncology care. Age at diagnosis was the only continuous covariate.

**Table 1:** Distribution of demographic variables according to race and ethnicity, comparing non-Hispanic White women with racial and ethnic minority women combined (*n* = 1836)

	Non-Hispanic White (*n* = 1350)	Racial and ethnic minority women (*n* = 486)	Total (*n* = 1836)	
Variable	*n* (%)	*n* (%)	*n* (%)	*p*
Country of origin*				< 0.0001
US Born	1,234 (91.5)	279 (57.4)	1,513 (82.5)	
Non US Born	114 (8.5)	207 (42.6)	321 (17.5)	
Age at diagnosis				< 0.0001
Years, (mean ± SD)	63.05 ± 10.87	57.09 ± 11.70	61.47 ± 11.40	
Education level				< 0.0001
High school or less	153 (11.3)	96 (19.8)	249 (13.6)	
Some college	453 (33.6)	144 (29.6)	597 (32.5)	
College graduate	366 (27.1)	153 (31.5)	519 (28.3)	
Post graduate	376 (27.9)	93 (19.1)	469 (25.5)	
Unknown	2 (0.1)	0 (0)	2 (0.1)	
Income level				0.83
< $25 K	105 (7.8)	51 (10.5)	156 (8.5)	
$25 K–$49 K	255 (18.9)	90 (18.5)	345 (18.8)	
$50 K–$89 K	419 (31.0)	137 (28.2)	556 (30.3)	
≥ $90 K	449 (33.3)	148 (30.5)	597 (32.5)	
Unknown	122 (9.0)	60 (12.3)	182 (9.9)	
Marital status				0.23
Married/lived as married	854 (63.3)	323 (66.5)	1,177 (64.1)	
Single/separated/widowed	492 (36.4)	161 (33.1)	653 (35.6)	
Unknown	4 (0.3)	2 (0.4)	6 (0.3)	
Provider race or ethnicity				0.64
White	550 (40.7)	207 (42.6)	757 (41.2)	
Asian	650 (48.1)	227 (46.7)	877 (47.8)	
Black	33 (2.4)	12 (2.5)	45 (2.5)	
Hispanic	42 (3.1)	12 (2.5)	54 (2.9)	
Unknown	75 (5.6)	28 (5.8)	103 (5.6)	

*n = 2 women with missing country of origin

### Statistical analysis

To ensure rigor and reproducibility, we assessed the agreement between respective question responses at baseline and at the follow-up time periods using Cohen’s Kappa statistics for percentage agreement. The percentage of observed agreements were measured in two dimensions: (1) ever versus never racial/ethnic discrimination in patient-provider interactions for the two questions at baseline and their respective follow-up responses in the Pathways Study Social Measure questionnaire; and (2) like previous, instead with the original five responses to the two questions and their respective follow-ups. The observed agreements were above 90% in all combinations for self-reported racial/ethnic discrimination in patient-provider interaction variables and follow-ups. Overall, the responses to these two questions at baseline and the two follow-up time points are consistent in all dimensions with a high level of agreement (Supplement Table 2).

**Table 2:** Distribution of demographic variables as potential confounders stratified by ever or never self-reported racial or ethnic discrimination in patient-provider interactions (*n* = 1836)

	Never SRREDPPI (*n* = 1584)	Ever SRREDPPI (*n* = 252)	Total (*n* = 1836)	
Variable	*n* (%)	*n* (%)	*n* (row %)	*p*
Race/ethnicity				< 0.0001
Non-Hispanic White	1,244 (92.1)	106 (7.9)	1,350 (100)	
Racial and ethnic minority	340 (70.0)	146 (30.0)	486 (100)	
Race/ethnicity strata				< 0.0001
Non-Hispanic White	1,244 (92.1)	106 (7.9)	1,350 (100)	
Black	48 (55.2)	39 (44.8)	87 (100)	
Asian	137 (65.9)	71 (34.1)	208 (100)	
Hispanic	122 (79.7)	31 (20.3)	153 (100)	
American Indian, Alaska Native and Pacific Islander	33 (86.8)	5 (13.2)	38 (100)	
Country of origin*				< 0.0001
US born	1,344 (88.8)	169 (11.2)	1,513 (100)	
Non-US born	239 (74.5)	82 (25.5)	321 (100)	
Age at diagnosis				0.07
Years, (mean ± SD)	61.66 ± 11.28	60.25 ± 12.09	61.47 ± 11.40	
Education level				0.26
High school or less	213 (85.5)	36 (14.5)	249 (100)	
Some college	513 (85.9)	84 (14.1)	597 (100)	
College graduate	440 (84.8)	79 (15.2)	519 (100)	
Post graduate	417 (88.9)	52 (11.1)	469 (100)	
Unknown	1 (50.0)	1 (50.0)	2 (100)	
Income level				0.01
< $25 K	129 (82.7)	27 (17.3)	156 (100)	
$25 K–$49 K	283 (82.0)	62 (18.0)	345 (100)	
$50 K–$89 K	480 (86.3)	76 (13.7)	556 (100)	
≥ $90 K	534 (89.4)	63 (10.6)	597 (100)	
Unknown	158 (86.8)	24 (13.2)	182 (100)	
Marital status				0.01
Married/lived as married	1033 (87.8)	144 (12.2)	1,177 (100)	
Single/separated/widowed	547 (83.8)	106 (16.2)	653 (100)	
Unknown	4 (66.7)	2 (33.3)	6 (100)	
Provider race or ethnicity				0.97
White	652 (86.1)	105 (13.9)	757 (100)	
Asian	759 (86.5)	118 (13.5)	877 (100)	
Black	35 (77.8)	10 (22.2)	45 (100)	
Hispanic	52 (96.3)	2 (3.7)	54 (100)	
Unknown	86 (83.5)	17 (16.5)	103 (100)	

*SRREDPPI* Self-reported racial/ethnic discrimination in patient-provider interactions

*n = 2 women with missing country of origin

We performed descriptive univariate analyses comparing NHW vs. all minority groups combined and with individual race and ethnicity subgroups, examining demographic and clinical characteristics as frequencies and proportions for categorical variables or median and standard deviation (SD) for continuous variables. We used univariable logistic regression to calculate odds ratios and 95% confidence intervals for those with and without self-reported racial/ethnic discrimination in patient-provider interactions and NHW vs. minority women and additionally by race and ethnicity strata.

To assess whether associations of self-reported racial/ethnic discrimination in patient-provider interactions differed by race and ethnicity, we used multivariable logistic regression to calculate adjusted odds ratios and 95% confidence intervals. Two sets of models were performed. The first overall model examined the associations of self-reported racial/ethnic discrimination in patient-provider interactions by comparing all minority women combined with NHW women. The second models were stratified models by each racial and ethnic group of women comparing with NHW women. Models were adjusted for age at diagnosis, education level, income level, country of origin, marital status, and provider race or ethnicity. Additionally, we carried out a sensitivity analysis focused on each of the two questions independently and combined, based on their similarity and potential overlap. The sensitivity analysis estimated the odds ratios and *p*-value for each question independently and combined responses to the two questions (Supplemental Tables 3 and 4). All statistical analyses were performed in RStudio 1.2 (Rstudio Team, Boston, MA). Results were considered statistically significant when *p*-values were < 0.05; all tests were two-sided.

**Table 3:** Univariable and multivariable logistic regression models of the association of self-reported racial or ethnic discrimination in patient-provider interactions (SRREDPPI)* and follow-up responses by select variables of interest, including all racial and ethnic minority groups combined (*n* = 1836)

		Univariable models		Multivariable model^**^	
Variable	*n*	OR (95% CI)	*p*	aOR (95% CI)	*p*
Race and ethnicity					
Non-Hispanic White	1350	Ref		Ref	
Racial and ethnic minority	486	5.04 (3.82–6.66)	< 0.0001	4.73 (3.45–6.50)	< 0.0001
Country of origin***					
US born	1513	Ref		Ref	
Non-US born	321	2.73 (2.02–3.67)	< 0.0001	1.47 (1.03–2.07)	0.03
Age at diagnosis					
Years, (mean ± SD)	1836	0.99 (0.98–1.00)	0.07	1.00 (0.99–1.02)	0.74
Education level					
High school or less	249	Ref		Ref	
Some college	597	0.97 (0.64–1.49)	0.88	1.29 (0.83–2.04)	0.27
College graduate	519	1.06 (0.70–1.64)	0.78	1.34 (0.85–2.16)	0.22
Post graduate	469	0.74 (0.47–1.17)	0.19	1.17 (0.70–1.95)	0.55
Unknown	2	5.92 (0.23–151.89)	0.21		
Income level					
< $25 K	156	Ref		Ref	
$25 K–$49 K	345	1.05 (0.64–1.74)	0.86	1.28 (0.75–2.23)	0.37
$50 K–$89 K	556	0.76 (0.47–1.24)	0.25	0.99 (0.58–1.75)	0.98
≥ $90 K	597	0.56 (0.35–0.93)	0.02	0.75 (0.41–1.39)	0.36
Unknown	182	0.73 (0.40–1.32)	0.29	0.78 (0.40–1.51)	0.47
Marital status					
Married/lived as married	1177	Ref		Ref	
Single/separated/widowed	653	1.39 (1.06–1.82)	0.02	1.36 (0.98–1.87)	0.06
Unknown	6	3.59 (0.49–18.55)	0.14	1.63 (0.07–16.16)	0.70
Provider race or ethnicity					
White	757	Ref		Ref	
Asian	877	0.97 (0.73–1.28)	0.81	0.96 (0.71–1.30)	0.81
Black	45	1.77 (0.81–3.56)	0.13	1.82 (0.79–3.86)	0.14
Hispanic	54	0.24 (0.04–0.78)	0.05	0.24 (0.04–0.82)	0.06
Unknown	103	1.23 (0.68–2.10)	0.47	1.14 (0.61–2.04)	0.66

*Questions related to self-perceived discrimination are:

How often did doctors pay less attention to you because of your race or ethnicity?

How often did you feel discriminated against by doctors because of your race or ethnicity?

**Adjusted for covariates including country of origin, age, education level, income level, marital status, and provider race or ethnicity

***n = 2 women missing country of origin

**Table 4:** Univariable and multivariable logistic regression models of the association of self-reported racial or ethnic discrimination in patient-provider interactions (SRREDPPI)* and follow-up responses by select variables of interest, including race and ethnicity groups (*n* = 1836)

		Univariable Models		Multivariable Model**	
Variable	*n*	OR (95% CI)	*p*	aOR (95% CI)	*p*
Race and ethnicity					
Non-Hispanic White	1,350	Ref		Ref	
Black	87	9.54 (5.96–15.20)	< 0.001	9.65 (5.92–15.70)	< 0.0001
Asian	208	6.08 (4.29–8.61)	< 0.001	5.39 (3.46–8.40)	< 0.0001
Hispanic	153	2.98 (1.89–4.59)	< 0.001	2.55 (1.54–4.14)	0.0002
American Indian, Alaska Native, and Pacific Islander	38	1.78 (0.60–4.27)	< 0.001	1.74 (0.58–4.25)	0.27
Country of origin***					
US born	1513	Ref		Ref	
Non-US born	321	2.73 (2.02–3.67)	< 0.0001	1.62 (1.09–2.39)	0.01
Age at diagnosis					
Years, (mean ± SD)	1836	0.99 (0.98–1.00)	0.07	1.00 (0.99–1.02)	0.73
Education level					
High school or less	249	Ref		Ref	
Some college	597	0.97 (0.64–1.49)	0.88	1.21 (0.77–1.93)	0.42
College graduate	519	1.06 (0.70–1.64)	0.78	1.11 (0.69–1.83)	0.67
Post graduate	469	0.74 (0.47–1.17)	0.19	1.00 (0.60–1.70)	0.99
Unknown	2	5.92 (0.23–151.89)	0.21		
Income level					
< $25 K	156	Ref		Ref	
$25 K–$49 K	345	1.05 (0.64–1.74)	0.86	1.46 (0.85–2.59)	0.18
$50 K–$89 K	556	0.76 (0.47–1.24)	0.25	1.15 (0.66–2.05)	0.63
≥ $90 K	597	0.56 (0.35–0.93)	0.02	0.79 (0.43–1.49)	0.47
Unknown	182	0.73 (0.40–1.32)	0.29	0.87 (0.44–1.70)	0.68
Marital status					
Married/lived as married	1177	Ref		Ref	
Single/separated/widowed	653	1.39 (1.06–1.82)	0.02	1.27 (0.91–1.75)	0.16
Unknown	6	3.59 (0.49–18.54)	0.14	1.40 (0.06–14.14)	0.79
Provider race or ethnicity					
White	757	Ref		Ref	
Asian	877	0.97 (0.73–1.28)	0.81	0.98 (0.72–1.33)	0.91
Black	45	1.77 (0.81–3.56)	0.13	1.81 (0.78–3.87)	0.14
Hispanic	54	0.24 (0.04–0.78)	0.05	0.24 (0.04–0.83)	0.06
Unknown	103	1.23 (0.68–2.10)	0.47	1.06 (0.56–1.92)	0.85

*Questions related to self-perceived discrimination are:

How often did doctors pay less attention to you because of your race or ethnicity?

How often did you feel discriminated against by doctors because of your race or ethnicity?

**Adjusted for covariates including country of origin, age, education level, income level, marital status, and provider race or ethnicity

***n = 2 women missing country of origin

## Results

**Fig. 2:**
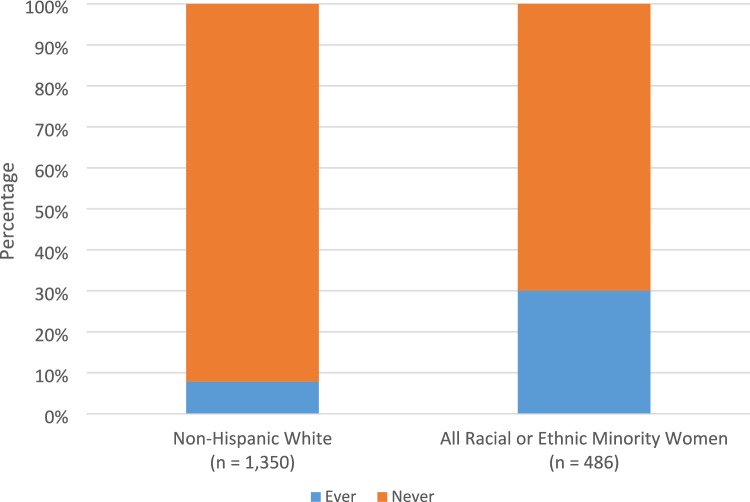
Distribution of ever or never self-reported racial/ethnic discrimination in patient-provider interactions (SRREDPPI) and follow-up responses stratified by non-Hispanic white vs. all racial or ethnic minority women (*n* = 1836)

Among the 1836 (1350 NHW and 486 women from race or ethnic minority groups) eligible women, minority women were more likely to report being paid less attention to or feeling discriminated against by doctors because of their race or ethnicity (30.0% vs. 7.9%) (Table 1 and Fig. 2). Minority women were more likely to be foreign-born, have a high school degree or less, and were younger at diagnosis than NHW women (Table 1). No differences were found in the distributions of income level, marital status, and provider race or ethnicity between NHW women and minority women.

When examining self-reported racial/ethnic discrimination in patient-provider interactions, women who reported racial or ethnic discrimination were more likely to be minorities, non-US born, earning less than $50 K per year, single/separated/widowed, and younger at diagnosis compared to those who did not report such discrimination (Table 2). There were no differences in the distribution of education level and provider race or ethnicity between those who reported discrimination and those who did not.

Of the sub-analyses examining individual race or ethnic groups, Black women were most likely to report being paid less attention to and feeling discriminated by doctors because of their race/ethnicity (44.8%), followed by Asian women (34.1%), Hispanic women (20.3%), AIANPI women (13.2%) and NHW women (7.9%).

**Fig. 3:**
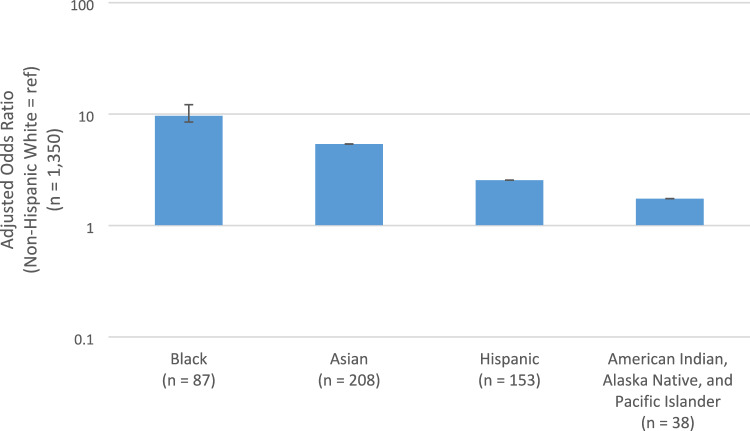
Multivariable logistic regression models of self-reported racial or ethnic discrimination in patient-provider interactions (SRREDPPI) and follow-up responses stratified by race and ethnic groups (*n* = 1836)

Results of the univariable and multivariable models were similar when examining minority women overall or stratified by race and ethnicity (Tables 3 and 4). In the multivariable model comparing minority women combined vs. NHW women, the odds of ever being paid less attention to and feeling discriminated by doctors because of race or ethnicity was significantly higher for minority women (adjusted odds ratio [aOR] 4.73, 95% CI 3.45–6.50) than their NHW counterparts. The multivariable model in the five race and ethnicity groups showed that each race or ethnic minority groups had higher odds of self-reported racial/ethnic discrimination in patient-provider interactions than NHW women (Black aOR: 9.65, 95% CI 5.92–15.70; Asian aOR: 5.39, 95% CI 3.46–8.40; Hispanic aOR: 2.55, 95% CI 1.54–4.14; and American Indian, Alaska Native, and Pacific Islander aOR: 1.74, 95% CI 0.58–4.25) (Fig. 3). In these models, being non-US born was statistically significantly associated with increases in self-reported racial or ethnic discrimination in patient-provider interactions, whereas having a Hispanic provider was associated with a decrease in self-reported discrimination.

Sensitivity analyses that independently compared estimates for each of the two questions independently rather than combined revealed that models comparing all minority women combined with NHW women, and stratified by race and ethnicity subgroup did not differ substantially in magnitude of odds ratios or statistical significance. Some differences were observed in responses to the two questions by some of the covariates. Women who were single/separated/widowed [model 1 aOR: 1.43 (95% CI 1.03–1.99), *p*-value 0.04; model 2 aOR: 1.30 (95% CI 0.95–1.79], *p*-value 0.10) had significant odds ratios only for being paid less attention by doctors. For race and ethnicity subgroups, no differences were found in response to the two questions. (Supplemental Tables 3 and 4).

## Discussion

The identification of factors contributing to the health inequity of Black women in the US having the highest age-adjusted breast cancer mortality rates compared with all other racial/ethnic groups is important and timely. Breast cancer of advanced stage, high grade, and hormone receptor (HR)-negative tumors are often the most frequent prognostic factors for the phenomenon; however, the role of racism, including interpersonal, institutional, and structural racism, is rarely considered and evaluated as a causal mechanism for these racial and ethnic disparities.

In this cohort study of 1836 women diagnosed with invasive breast cancer, among women who self-identified as Hispanic, Black, Asian, American Indian, Alaska Native, and Pacific Islander, a substantial proportion (30%) reported discrimination due to race or ethnicity in patient-provider interactions compared with NHW women (7.9%). Our findings of Black women reported the highest (45%) percentage of ever experiencing racial discrimination in patient-provider interactions was consistent in previous studies [11, 13, 18, 19]. Additionally, in multivariable models, we found that race and ethnicity groups continued to be associated with self-reported racial/ethnic discrimination in patient-provider interactions. Although these associations became somewhat attenuated after adjusting for covariates for Asian, Hispanic, and AIANPI women, the association remained significant and even strengthened in Black women. This suggests that, Black women frequently experience discrimination in their clinical encounters and may impact perceptions of care within the health care setting. Given our findings, a need remains to fully understand the mechanism of disparities in breast cancer mortality in Black women.

We hypothesized that women who self-identify as Black, Hispanic, Asian, American Indian, Alaska Native, and Pacific Islander would be more likely to self-report ever experiencing racial/ethnic discrimination in patient-provider interactions compared with their NHW cohort. This was based on the legacy of historical and contemporary racism in the U.S., including in medical settings. Experiences of racial or ethnic discrimination, in this capacity, has consistently been associated with adverse health outcomes and health disparities, including adverse pregnancy outcomes [20], insomnia disorder [21], and cardiovascular disease [22].

Differences in medical care and outcomes are complex and multifactorial. Studies in health care systems have suggested that people who identify as a racial or ethnic minority receive poorer quality of health care than people who identify as NHW, even after adjustment for sociodemographic factors and payment by the patient [23]. Other studies have also found that self-reported discrimination due to race or ethnicity in healthcare settings is associated with delays in cancer screenings [24, 25], tests or treatments prescribed by their healthcare provider [26, 27], and low patient satisfaction [11, 28, 29]. Such discrimination in healthcare settings is perpetuated by institutional policies and conscious and unconscious biases based on stereotypes of racial and ethnic minorities. For example, one study found that white medical students were more likely to believe that Black people had a higher tolerance for pain than white people [30]. This study of 222 white medical students found that about half of all participants endorsed at least one false belief about biological differences in pain between Black and whites [30]. Some false beliefs included in this study were that Black people had thicker skin, stronger immune systems, and nerve endings that were less sensitive than whites [30].

Although most physicians who operate in these healthcare settings believe that they are not explicitly racist and treat all patients equally, many consider the system they work in is inherently racist [31]. Racial discrimination patterns and practices by office staff, specifically providers, may likely contribute to medical mistrust, and could lead to healthcare disparities in breast cancer outcomes [32–34]. Studies have speculated that medical mistrust may be a product of systemic racism [35], thereby impacting adverse cancer outcomes in Black patients [32, 33]. Addressing racism in the form of racial or ethnic discrimination in patient-provider interactions with efforts, such as implicit bias and antiracism training, would improve the quality of patient-provider relationships and inform interventions to ameliorate disparities in breast cancer outcomes.

Our study has several strengths. The Pathways Study is a racially and ethnically diverse breast cancer survivorship cohort with measures at baseline and through follow-up periods that allowed us to engage women who self-reported racial/ethnic discrimination in patient-provider interactions. The multiple longitudinal measurements of self-reported racial/ethnic discrimination in patient-provider interactions in the IPC survey enabled evaluation of the exposure over cross-sectional time points, expanding upon previous findings limited by a cross-sectional design. Another strength is the recruitment of study participants in the KPNC healthcare system. The recruitment was advantageous in collecting many lifestyle factors at baseline around two months post-diagnosis, allowing measures of patient-provider interactions soon after diagnosis and the potential absence of survival bias. Lastly, this study collected validated measures of self-reported racial/ethnic discrimination in patient-provider interactions through the IPC survey, which has been validated in diverse and racial and ethnic backgrounds [13].

Our study also had some limitations. First, the racial/ethnic discrimination outcome measure was based on two questions from the IPC survey, unlike full scales that measure discrimination aptly in medical settings relating to treatment and perceptions. Despite the limitation, the wording of one of the two questions of the IPC survey was direct and attributable to the patient’s race or ethnicity, which some scales did not assess. Second, the timing and specific care provider(s) were not detailed in how the racial or ethnic discrimination questions were queried. The IPC questionnaire refers to interactions with “your doctors over the past 12 months.” For the purposes of identifying the race or ethnicity of the breast cancer patients’ providers, we assumed that interactions referred to the medical oncologist as the first provider of choice, and other cancer care providers otherwise. Indeed, the medical oncologist was identified as the provider for nearly 95% of study participants. However, responses may also have been answered with participants considering other providers, including those who may not be involved in their cancer care. Third, despite significant associations for sub-analyses that stratified by racial and ethnic strata, minority populations had smaller sample sizes than NHW women. Thus, inferences from racial or ethnic minority analyses should be made with considerable caution. Last, our sample was drawn from the Pathways Study of breast cancer patients diagnosed and treated between 2006 and 2013, which was conducted in the Kaiser Permanente Northern California health care setting, and thus limited to insured patients with managed healthcare plans. Therefore, results may not be applicable to other health care systems or to uninsured patients. Nevertheless, this includes well-developed virtual healthcare systems, involving video conferencing and online services, our findings are consistent with those from other studies examining racial or ethnic discrimination in health care.

In summary, in a large, diverse prospective cohort study of breast cancer survivors with data collected over two years, we found that women from self-identified racial or ethnic minority groups were more likely to self-report racial/ethnic discrimination in patient-provider interactions than NHW women. This association was observed in racial/ethnic minority groups combined and in each racial or ethnic group separately, and remained after accounting for differences in sociodemographic and oncology provider’s race and ethnicity. These findings underscore the potential significance of addressing racial/ethnic minority women’s experiences of self-reported discrimination in cancer survivors. The specific mechanisms through which racial and ethnic discrimination arises in the health care setting, and how this may result in adverse outcomes in cancer patients, should be pursued. Future analyses in this cohort may address whether self-reported racial/ethnic discrimination impacts treatments received and breast cancer outcomes. Finally, with this knowledge, interventions to address and mitigate self-reported racial/ethnic discrimination in patient-provider interactions may be appropriate for development and efficacy/effectiveness testing.

## Supplementary information

Below is the link to the electronic supplementary material.

Supplementary file1 (DOCX 157 kb)

## Data Availability

The datasets generated during and/or analyzed during the current study are not publicly available due to patient confidentiality but are available from the Pathways Study authors on reasonable request.
