# Supplementary material for: Race and ethnicity and self-reported racial/ethnic discrimination in breast cancer patient interactions with providers in the Pathways Study
Source: Breast Cancer Res Treat. 2024 Oct 5;209(2):355–66. doi: 10.1007/s10549-024-07499-0 (PMC11785627; doi:10.1007/s10549-024-07499-0)
Supplement: Supplementary file 1 — Supplementary file1 (DOCX 157 kb) [file 10549_2024_7499_MOESM1_ESM.docx]

Supplemental Table 1. Distribution of sociodemographic variables according to race and ethnicity, comparing non-Hispanic White women with racial and ethnic minority women combined based on missing self-reported racial or ethnic discrimination in patient-provider interaction (SRREDPPI) variables and follow-up responses (n = 4,504)

| \|  \| Excluded in analysis \| \| \| Included in analysis \| \| \| \| --- \| --- \| --- \| --- \| --- \| --- \| --- \| \|  \| Non-Hispanic White women  (n =1,600) \| All racial or ethnic minority women  (n =1,068) \| Total  (n = 2,668) \| Non-Hispanic White women  (n = 1,350) \| All racial or ethnic minority women  (n = 486) \| Total  (n = 1,836) \| \| Variable \| n (%) \| n (%) \| n (%) \| n (%) \| n (%) \| n (%) \| \| Country of origin* \|  \|  \|  \|  \|  \|  \| \| Non-US \| 137 (8.6) \| 478 (44.8) \| 615 (23.1) \| 114 (8.5) \| 207 (42.6) \| 321 (17.5) \| \| US Born \| 1459 (91.2) \| 587 (55.0) \| 2046 (76.7) \| 1234 (91.5) \| 279 (57.4) \| 1513 (82.4) \| \| Age at diagnosis (years) \|  \|  \|  \|  \|  \|  \| \| < 50 \| 295 (18.4) \| 386 (36.1) \| 681 (25.5) \| 157 (11.6) \| 141 (29.0) \| 298 (16.2) \| \| 50 – 59 \| 478 (29.9) \| 380 (35.6) \| 858 (32.2) \| 314 (23.3) \| 135 (27.8) \| 449 (24.5) \| \| 60 – 69 \| 465 (29.1) \| 188 (17.6) \| 653 (24.5) \| 500 (37.0) \| 139 (28.6) \| 639 (34.8) \| \| ≥ 70 \| 359 (22.4) \| 111 (10.4) \| 470 (17.6) \| 377 (27.9) \| 71 (14.6) \| 448 (24.4) \| \| Unknown \| 3 (0.2) \| 3 (0.3) \| 6 (0.2) \| 2 (0.1) \| 0 (0) \| 2 (0.1) \| \| Education level \|  \|  \|  \|  \|  \|  \| \| HS or less \| 227 (14.2) \| 231 (21.6) \| 458 (17.2) \| 153 (11.3) \| 96 (19.8) \| 249 (13.6) \| \| Some College \| 587 (36.7) \| 383 (35.9) \| 970 (36.4) \| 453 (33.6) \| 144 (29.6) \| 597 (32.5) \| \| College Grad \| 415 (25.9) \| 307 (28.7) \| 722 (27.1) \| 366 (27.1) \| 153 (31.5) \| 519 (28.3) \| \| Post Grad \| 368 (23.0) \| 144 (13.5) \| 512 (19.2) \| 376 (27.9) \| 93 (19.1) \| 469 (25.5) \| \| Unknown \| 3 (0.2) \| 3 (0.3) \| 6 (0.2) \| 2 (0.1) \| 0 (0) \| 2 (0.1) \| \| Income level \|  \|  \|  \|  \|  \|  \| \| < $25K \| 157 (9.8) \| 120 (11.2) \| 277 (10.4) \| 105 (7.8) \| 51 (10.5) \| 156 (8.5) \| \| $25K – $49K \| 296 (18.5) \| 203 (19.0) \| 499 (18.7) \| 255 (18.9) \| 90 (18.5) \| 345 (18.8) \| \| $50K - $89K \| 421 (26.3) \| 304 (28.5) \| 725 (27.2) \| 419 (31.0) \| 137 (28.2) \| 556 (30.3) \| \| ≥ $90K \| 535 (33.4) \| 278 (26.0) \| 813 (30.5) \| 449 (33.3) \| 148 (30.5) \| 597 (32.5) \| \| Unknown \| 191 (11.9) \| 163 (15.3) \| 354 (13.3) \| 122 (9.0) \| 60 (12.3) \| 182 (9.9) \| \| Marital Status \|  \|  \|  \|  \|  \|  \| \| Married/Lived as Married \| 916 (57.3) \| 644 (60.3) \| 1560 (58.5) \| 854 (63.3) \| 323 (66.5) \| 1177 (64.1) \| \| Single/Sep/Wid \| 681 (42.6) \| 417 (39.0) \| 1098 (41.2) \| 492 (36.4) \| 161 (33.1) \| 653 (35.6) \| \| Unknown \| 3 (0.2) \| 7 (0.7) \| 10 (0.4) \| 4 (0.3) \| 2 (0.4) \| 6 (0.3) \| \| Provider race or ethnicity \|  \|  \|  \|  \|  \|  \| \| Asian \| 741 (46.3) \| 545 (51.0) \| 1286 (48.2) \| 650 (48.1) \| 227 (46.7) \| 877 (47.8) \| \| Black \| 31 (1.9) \| 9 (0.8) \| 40 (1.5) \| 33 (2.4) \| 12 (2.5) \| 45 (2.5) \| \| Hispanic \| 33 (2.1) \| 33 (3.1) \| 66 (2.5) \| 42 (3.1) \| 12 (2.5) \| 54 (2.9) \| \| White \| 709 (44.3) \| 411 (38.5) \| 1120 (42.0) \| 550 (40.7) \| 207 (42.6) \| 757 (41.2) \| \| Unknown \| 86 (5.3) \| 70 (6.6) \| 156 (5.8) \| 75 (5.6) \| 28 (5.8) \| 103 (5.6) \| |
| --- | --- | --- | --- | --- | --- | --- | --- | --- | --- | --- | --- | --- | --- | --- | --- | --- | --- | --- | --- | --- | --- | --- | --- | --- | --- | --- | --- | --- | --- | --- | --- | --- | --- | --- | --- | --- | --- | --- | --- | --- | --- | --- | --- | --- | --- | --- | --- | --- | --- | --- | --- | --- | --- | --- | --- | --- | --- | --- | --- | --- | --- | --- | --- | --- | --- | --- | --- | --- | --- | --- | --- | --- | --- | --- | --- | --- | --- | --- | --- | --- | --- | --- | --- | --- | --- | --- | --- | --- | --- | --- | --- | --- | --- | --- | --- | --- | --- | --- | --- | --- | --- | --- | --- | --- | --- | --- | --- | --- | --- | --- | --- | --- | --- | --- | --- | --- | --- | --- | --- | --- | --- | --- | --- | --- | --- | --- | --- | --- | --- | --- | --- | --- | --- | --- | --- | --- | --- | --- | --- | --- | --- | --- | --- | --- | --- | --- | --- | --- | --- | --- | --- | --- | --- | --- | --- | --- | --- | --- | --- | --- | --- | --- | --- | --- | --- | --- | --- | --- | --- | --- | --- | --- | --- | --- | --- | --- | --- | --- | --- | --- | --- | --- | --- | --- | --- | --- | --- | --- | --- | --- | --- | --- | --- | --- | --- | --- | --- | --- | --- | --- | --- | --- | --- | --- | --- | --- | --- | --- | --- | --- | --- | --- | --- | --- | --- | --- | --- | --- | --- | --- | --- | --- | --- | --- | --- | --- | --- | --- | --- | --- | --- | --- | --- | --- | --- | --- | --- | --- |

* n = 7 women missing country of origin in the excluded analysis and n = 2 missing country of origin in the included analysis

Supplemental Table 2. Agreement of responses for ever/never and original responses of self-reported racial or ethnic discrimination in patient-provider interaction (SRREDPPI)* variables and their respective follow-up responses (6-months and 24-months) among Pathways Study participants (primary analysis and sub-analysis n = 1836)

| \| Type of analysis \| Ever/never responses to the two questions \| Percentage of Observed Agreements \| Original five responses to the two questions  (never, rarely, sometimes, usually, always) \| Percentage of Observed Agreements \| \| --- \| --- \| --- \| --- \| --- \| \| Primary and sub-analysis (all racial or ethnic minority women vs. NHW) \| Q1 baseline and 6-months \| 91.5% \| Q1 baseline and 6-months \| 90.9% \| \|  \| Q1 baseline and 24-months \| 91.2% \| Q1 baseline and 24-months \| 90.5% \| \|  \| Q1 6-months and 24-months \| 93.8% \| Q1 6-months and 24-months \| 93.3% \| \|  \| Q2 baseline and 6-months \| 94.0% \| Q2 baseline and 6-months \| 93.7% \| \|  \| Q2 baseline and 24-months \| 95.0% \| Q2 baseline and 24-months \| 94.4% \| \|  \| Q2 6-months and 24-months \| 95.8% \| Q2 6-months and 24-months \| 95.4% \| |
| --- | --- | --- | --- | --- | --- | --- | --- | --- | --- | --- | --- | --- | --- | --- | --- | --- | --- | --- | --- | --- | --- | --- | --- | --- | --- | --- | --- | --- | --- | --- | --- | --- | --- | --- | --- |

* Questions related to self-perceived discrimination are:

Q1: How often did doctors pay less attention to you because of your race or ethnicity?

Q2: How often did you feel discriminated against by doctors because of your race or ethnicity?

Supplemental Table 3. Comparison of “Q1: How often did doctors pay less attention to you because of your race or ethnicity?” and “Q2: How often did you feel discriminated against by doctors because of your race or ethnicity?” and follow-up responses by all racial or ethnic minority groups vs. NHW women (Q1 n = 1838; Q2 n = 1854)

| \|  \| Model 1 (Q1) \| Model 2 (Q2) \| \| --- \| --- \| --- \| \|  \| aOR (95% CI)  p-value \| aOR (95% CI)  p-value \| \| Racial and ethnic minority \| 5.11 (3.70 – 7.07)  < 0.0001 \| 4.63 (3.39 – 6.34)  < 0.0001 \| \| Non-US Born \| 1.52 (1.07 – 2.15)  0.02 \| 1.50 (1.06 – 2.10)  0.02 \| \| Age at diagnosis, continuous \| 1.01 (0.99 – 1.02)  0.47 \| 1.01 (1.00 – 1.02)  0.21 \| \| Some College \| 1.35 (0.86 – 2.17)  0.20 \| 1.37 (0.88 – 2.18)  0.17 \| \| College Graduate \| 1.29 (0.80 – 2.10)  0.30 \| 1.37 (0.86 – 2.22)  0.19 \| \| Post Graduate \| 1.17 (0.70 – 1.98)  0.56 \| 1.39 (0.84 – 2.31)  0.20 \| \| $25K - $49K \| 1.13 (0.65 – 1.98)  0.67 \| 1.23 (0.73 – 2.13)  0.45 \| \| $50K - $89K \| 1.00 (0.58 – 1.77)  0.99 \| 0.97 (0.57 – 1.70)  0.92 \| \| ≥ $90K \| 0.81 (0.44 – 1.51)  0.49 \| 0.75 (0.42 – 1.38)  0.35 \| \| Unknown \| 0.76 (0.39 – 1.49)  0.43 \| 0.84 (0.44 – 1.59)  0.59 \| \| Single/Separated/Widowed \| 1.43 (1.03 – 1.99)  0.04 \| 1.30 (0.95 – 1.79)  0.10 \| \| Unknown \| 1.73 (0.08 – 17.31)  0.66 \| 1.63 (0.07 – 16.47)  0.70 \| \| Provider race or ethnicity (Asian) \| 0.95 (0.70 – 1.30)  0.76 \| 0.92 (0.68 – 1.24)  0.57 \| \| Provider race or ethnicity (Black) \| 1.98 (0.86 – 4.21)  0.09 \| 1.99 (0.90 – 4.11)  0.07 \| \| Provider race or ethnicity (Hispanic) \| 0.26 (0.04 – 0.89)  0.07 \| 0.24 (0.04 – 0.81)  0.05 \| \| Provider race or ethnicity (Unknown) \| 1.21 (0.65 – 2.16)  0.54 \| 1.11 (0.61 – 1.95)  0.72 \| |
| --- | --- | --- | --- | --- | --- | --- | --- | --- | --- | --- | --- | --- | --- | --- | --- | --- | --- | --- | --- | --- | --- | --- | --- | --- | --- | --- | --- | --- | --- | --- | --- | --- | --- | --- | --- | --- | --- | --- | --- | --- | --- | --- | --- | --- | --- | --- | --- | --- | --- | --- | --- | --- | --- | --- |

Supplemental Table 4. Comparison of “Q1: How often did doctors pay less attention to you because of your race or ethnicity?” and “Q2: How often did you feel discriminated against by doctors because of your race or ethnicity?” and follow-up response by primary race and ethnicity-specific analysis odds ratio and p-value (Q1 n = 1838; Q2 n = 1854)

| \|  \| Model 1 (Q1) \| Model 2 (Q2) \| \| --- \| --- \| --- \| \|  \| aOR  (95% CI)  p-value \| aOR  (95% CI)  p-value \| \| Black \| 10.70 (6.54 – 17.49)  < 0.0001 \| 16.15 (9.26 – 28.10)  < 0.0001 \| \| Asian \| 5.75 (3.66 – 9.04)  <0.0001 \| 7.90 (4.64 – 13.50)  <0.0001 \| \| Hispanic \| 2.86 (1.72 – 4.66)  <0.0001 \| 4.21 (2.28 – 7.61)  <0.0001 \| \| American Indian, Alaska Native, and Pacific Islander \| 1.49 (0.43 – 3.90)  0.47 \| 2.66 (0.62 – 7.89)  0.12 \| \| Non-US Born \| 1.69 (1.13 – 2.50)  0.01 \| 1.66 (1.04 – 2.64)  0.03 \| \| Age at diagnosis, continuous \| 1.01 (0.99 – 1.02)  0.44 \| 0.99 (0.98 – 1.01)  0.40 \| \| Some College \| 1.29 (0.81 – 2.08)  0.29 \| 1.06 (0.60 – 1.91)  0.83 \| \| College Graduate \| 1.08 (0.66 – 1.80)  0.77 \| 1.32 (0.73 – 2.42)  0.36 \| \| Post Graduate \| 1.01 (0.59 – 1.74)  0.97 \| 1.14 (0.60 – 2.19)  0.70 \| \| $25K - $49K \| 1.30 (0.74 – 2.31)  0.37 \| 0.91 (0.47 – 1.79)  0.78 \| \| $50K - $89K \| 1.17 (0.67 – 2.10)  0.59 \| 0.78 (0.40 – 1.55)  0.47 \| \| ≥ $90K \| 0.86 (0.46 – 1.63)  0.65 \| 0.53 (0.26 – 1.12)  0.09 \| \| Unknown \| 0.85 (0.43 – 1.68)  0.65 \| 0.61 (0.27 – 1.35)  0.23 \| \| Single/Separated/Widowed \| 1.33 (0.95 – 1.86)  0.09 \| 1.02 (0.67 – 1.53)  0.92 \| \| Unknown \| 1.50 (0.07 – 15.24)  0.75 \| 0.000003 (NA – NA)  0.97 \| \| Provider race or ethnicity (Asian) \| 0.97 (0.71 – 1.33)  0.85 \| 1.17 (0.80 – 1.71)  0.42 \| \| Provider race or ethnicity (Black) \| 1.96 (0.84 – 4.21)  0.10 \| 0.70 (0.16 – 2.20)  0.58 \| \| Provider race or ethnicity (Hispanic) \| 0.26 (0.04 – 0.90)  0.07 \| 0.45 (0.07 – 1.61)  0.29 \| \| Provider race or ethnicity (Unknown) \| 1.13 (0.59 – 2.05)  0.70 \| 1.17 (0.53 – 2.41)  0.68 \| |
| --- | --- | --- | --- | --- | --- | --- | --- | --- | --- | --- | --- | --- | --- | --- | --- | --- | --- | --- | --- | --- | --- | --- | --- | --- | --- | --- | --- | --- | --- | --- | --- | --- | --- | --- | --- | --- | --- | --- | --- | --- | --- | --- | --- | --- | --- | --- | --- | --- | --- | --- | --- | --- | --- | --- | --- | --- | --- | --- | --- | --- | --- | --- | --- |

Supplemental Figure 1. Recruitment of the Pathways Study (N = 4,504) [36]

| 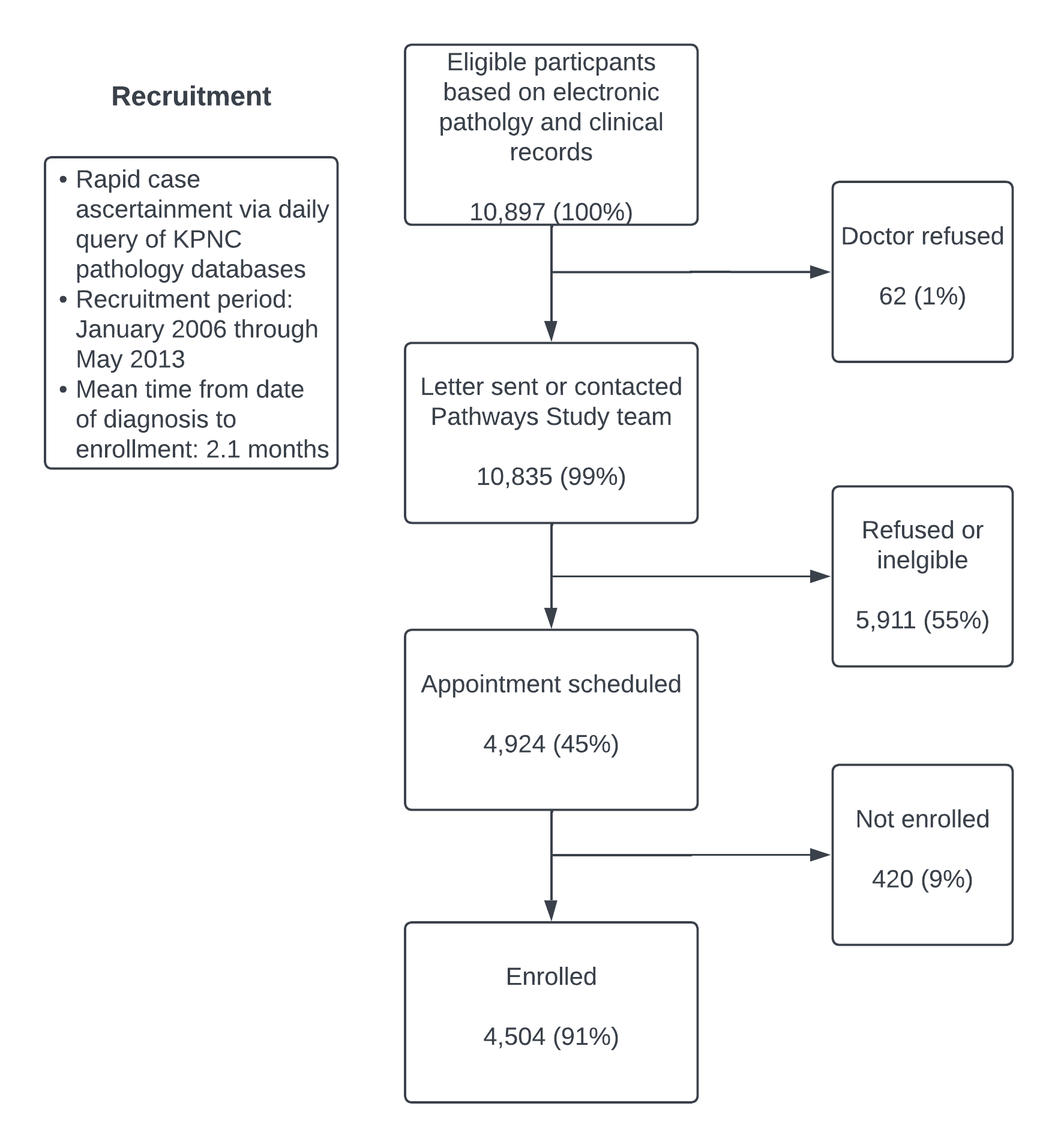 |
| --- |
